# Underwater Hyperspectral Imaging Technology and Its Applications for Detecting and Mapping the Seafloor: A Review

**DOI:** 10.3390/s20174962

**Published:** 2020-09-02

**Authors:** Bohan Liu, Zhaojun Liu, Shaojie Men, Yongfu Li, Zhongjun Ding, Jiahao He, Zhigang Zhao

**Affiliations:** 1 Key Laboratory of Laser & Infrared System, Ministry of Education, Shandong University, Qingdao 266237, China; liu_bh@mail.sdu.edu.cn (B.L.); zhaojunliu@sdu.edu.cn (Z.L.); yfli@sdu.edu.cn (Y.L.); zhigang@sdu.edu.cn (Z.Z.); 2 School of Information Science & Engineering and Shandong Provincial Key Laboratory of Laser Technology and Application, Shandong University, Qingdao 266237, China; jh_he@mail.sdu.edu.cn; 3 Center for Optics Research and Engineering, Shandong University, Qingdao 266237, China; 4 National Deep Sea Center, No.6, Xianxialing Road, Qingdao 266061, China; dzj@ndsc.org.cn

**Keywords:** underwater hyperspectral imaging, multispectral, marine mineral exploration, benthic habitat mapping, underwater archaeology

## Abstract

Common methods of ocean remote sensing and seafloor surveying are mainly carried out by airborne and spaceborne hyperspectral imagers. However, the water column hinders the propagation of sunlight to deeper areas, thus limiting the scope of observation. As an emerging technology, underwater hyperspectral imaging (UHI) is an extension of hyperspectral imaging technology in air conditions, and is undergoing rapid development for applications in shallow and deep-sea environments. It is a close-range, high-resolution approach for detecting and mapping the seafloor. In this paper, we focus on the concepts of UHI technology, covering imaging systems and the correction methods of eliminating the water column’s influence. The current applications of UHI, such as deep-sea mineral exploration, benthic habitat mapping, and underwater archaeology, are highlighted to show the potential of this technology. This review can provide an introduction and overview for those working in the field and offer a reference for those searching for literature on UHI technology.

## 1. Introduction

Hyperspectral imaging is a promising spectral imaging method based on reflection characteristics, which can provide a three-dimensional dataset of scenes, including the information of two spatial dimensions and one spectral dimension [1]. The spectral dimension of the hyperspectral dataset reflects the spectral information in the projection on the plane of the space. Therefore, hyperspectral imaging not only describes the two-dimensional geometry of the observed object, but can also be used to analyze and recognize the spectral characteristics. In the past few decades, hyperspectral imaging technology has been successfully applied to remote sensing studies of the Earth’s surface for land mineral exploration [2,3], precision agriculture [4,5], water management [6,7], and environmental surveys [8,9]. In addition, hyperspectral imaging is an effective tool in the field of marine research, such as ocean color remote sensing [10,11,12], coastal environmental investigations [13,14,15], disaster or pollution monitoring [16,17,18], and marine ecological surveys [19,20,21]. Currently, hyperspectral imaging based on airborne and spaceborne platforms has been able to observe targets covering the sea surface and within tens of meters underwater.

In recent years, with the development of ocean engineering and technologies, hyperspectral imaging is becoming more capable of taking images of water and of deeper underwater areas [22]. Underwater hyperspectral imaging (UHI) in applications of underwater environmental investigations have induced the interest of many researchers. With its high spatial and spectral resolution, UHI is capable of identifying and classifying objects on a fine scale. On the other hand, underwater hyperspectral data can be gathered based on manned and unmanned underwater vehicles to achieve a large-scale underwater investigation [23]. Therefore, UHI provides a new way to produce high-quality descriptions of underwater sediments, minerals, benthic habitats, and heritage sites [24], and can be used in further researches areas, such as deep-sea mineral exploration [25], benthic habitat mapping [26], and underwater archaeology [27].

Some authors have previously already mentioned the topics of hyperspectral imaging technology, but to the best of our knowledge, few reviews concerning UHI technology have been reported. In 2013, Johnsen et al. [24] introduced several aspects of UHI technology, such as optical fingerprints, georeferenced aspects, etc., and gave case examples of underwater carrying platforms and associated applications in the mapping of seafloor biogeochemical properties. In 2018, Lodhi et al. [23] also mentioned the usage of hyperspectral imaging on underwater platforms in a review of hyperspectral imaging on multiple platforms for earth observations.

The focus of this article is introducing and explaining UHI technology and providing an overview of the literature on the UHI system, correction methods of eliminating the water column’s influence, and applications in underwater investigation. We start with the mechanisms of UHI and its current development status. Then UHI systems are described based on the necessary components, including the imager, light source, and sensors. A consideration of the water’s influence in UHI processing is summarized, with an emphasis on correction and modeling methods. The part on applications is a reference to the literature available on marine mineral exploration, benthic habitat mapping, underwater archaeology, and pipeline inspection. Finally, we discuss application scenes of typical UHI systems and attempt to assess the future trend of UHI technology in the field of underwater applications.

## 2. Underwater Hyperspectral Imaging System

This section presents the three parts of the UHI system required for seafloor mapping: underwater hyperspectral imagers, a light source, and necessary sensors.

### 2.1. Underwater Hyperspectral Imager

An underwater hyperspectral imager is used to obtain the spectral characteristics and spatial distributions of objects for the seafloor mapping. Protection of the hyperspectral imager is provided by the pressure housing, which has an optical glass window for light transmission. The pressure housing is commonly made of aluminum alloy or stainless steel in shallow areas, while it is constructed of titanium alloy in deep-sea areas [28]. The cable connectors are used to provide energy and communications to the hyperspectral imager by connecting it with underwater vehicles.

**Figure 1:**
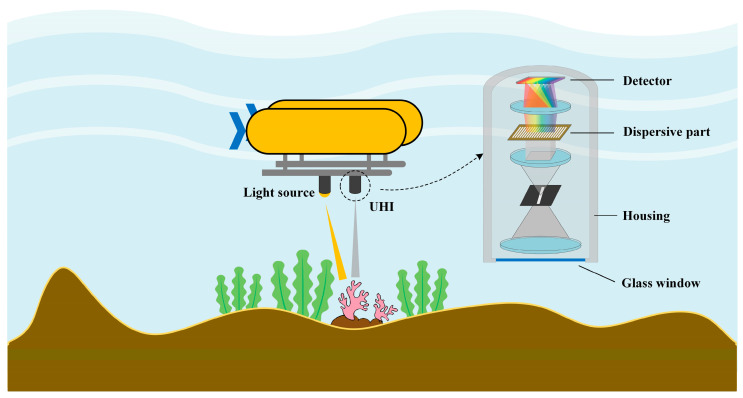
A schematic diagram of underwater hyperspectral imaging (UHI) for seafloor mapping.

The hyperspectral imager is the core component of the UHI system. Commonly, a hyperspectral imager consists of fore-optics, collimating optics, dispersive elements, focusing optics, and detectors. Light from an underwater scene is collected and collimated, respectively, by fore-optics and collimating optics, and then introduced into dispersive elements. The dispersive elements mainly include prisms, gratings, filters, and interferometers. After light splitting, a linear or plane array detector can be used to capture images. A schematic diagram of the operation and system framework of UHI is shown in Figure 1.

A few underwater spectral imagers have been reported, including multispectral and hyperspectral systems. In the case of multispectral systems, one of the earliest was that by Zawada [29], who developed a low-light-level underwater multispectral imaging system (LUMIS) for observing the fluorescence of marine organisms. LUMIS can capture four bands with narrow bandpass filters. With proprietary optics, the four channel images can be acquired simultaneously without any beam splitters. Gleason et al. [30] introduced a six-band filter wheel underwater multispectral camera for coral reef monitoring and automated classification. Wu et al. [31] presented an underwater spectral imaging system (UMSI) based on 31 narrowband color filters installed on double filter wheels. Double filter wheels can accommodate more color filters and reduce the system size. All of the above systems use filters as the dispersive elements; however, there are also some multispectral systems based on other considerations. For example, Liu et al. [32] reported a tunable LED-based underwater multispectral imaging system (TuLUMIS). TuLUMIS is an eight-band system, consisting of a monochrome camera and tunable LED. Without any dispersive element, images of all eight channels can be captured under the illumination of a combination of different wavelength LEDs. Moreover, Song et al. [33] developed an underwater multispectral system based on a liquid crystal tunable filter (LCTF), whose spectral resolution is 10 nm at a spectral range of 400–700 nm.

For hyperspectral systems, Johnsen [34] from the Norwegian University of Science and Technology (NTNU) invented the first UHI system. The divers placed the UHI system on a man-made underwater carrying cart with a cart-track on the seafloor to observe the objects in shallow water with the illumination of halogen lamps. After that, researchers at NTNU and Ecotone AS jointly developed a series of push-broom underwater hyperspectral imagers. In one commercially available version, the spectral resolution can reach up to 2.2 nm in 380–750 nm, with a grating as the dispersive element. What is impressive is that this system has a large field of view (~60°) along the cross-track direction and the immersion depth can reach up to 6000 m under pressure housing made of titanium alloy. Other companies, such as Cubert Gmbh and Sphere Optics, have also launched snapshot commercial underwater hyperspectral imagers. Lee et al. [35] reported two types of hyperspectral imagers. One was a hand-held imager operated by divers, and the other was a ship-towed system. Both of them were used for monitoring the health status of seagrass and coral reefs in shallow water areas.

Some studies built underwater hyperspectral systems by modifying and refitting the off-the-shelf imagers. For instance, Chennu et al. [36] designed an underwater hyperspectral system called HyperDiver. The core component of HyperDiver is a commercial hyperspectral imager (Pika 2, Resonon Inc., Bozeman, MT, USA). HyperDiver is a diver-operable push-broom system equipped with several sensors for monitoring water information. Nevala et al. [37] proposed a hyperspectral imager consisting of a commercial spectrometer and camera. Two rotating mirrors were used for scanning under a preset program and introduced light from the scanned scene into the spectrometer. Then, the spectrum and images captured by the camera were combined into hyperspectral images. Furthermore, some researchers have also reported the optical design of an underwater hyperspectral imager [38], which needs to be carefully designed in terms of the structure and size. The air-glass-water interface introduced by the optical glass window in front of the imager should also be considered in order to avoid a loss of performance [39,40]. Table 1 presents a comparison of some existing underwater spectral imaging systems.

As the key parameters of a hyperspectral imager, the spatial and spectral resolution depend on the performance of dispersive elements and the number of pixels in an array detector. A higher resolution will enrich the information level of objects, but increase the data size and bring challenges to data storage, transmission, and processing. In addition, the radiometric resolution of a hyperspectral imager should also be considered to ensure the high dynamic range of a system due to the brightness difference of objects.

### 2.2. Light Source

For optically shallow water areas, the UHI system works in a passive mode with solar illumination. However, when the UHI system acts in deep-sea areas and cannot get enough illumination, an active light source is necessary.

The light source is an indispensable part of the UHI system. Several visible light sources can be used in underwater environments, including halogen lamps, LEDs, and lasers [41,42,43]. However, there is currently no light source that is specifically designed for the UHI system. The reasons for this are diverse, and there are many special requirements for the light source used by UHI. The light sources used for UHI are broad spectrum, usually covering 400–700 nm or more in terms of spectral bands. In order to ensure that the detector collects enough photons reflected by the targets, the light source should have a sufficient intensity to illuminate underwater targets. The intensity in near-infrared and near-ultraviolet bands should be enhanced properly due to the high absorption in these ranges caused by the water column. Generally speaking, the height between the UHI system and the seafloor is usually 1–5 m (or nearly 10 m in extreme cases), thus, the light source is required to have a matching intensity. The light sources also need to have the advantages of high stability, high energy efficiency, and long life. In addition, a smaller size and lighter weight are equally important for ensuring that underwater vehicles can carry more loads, so as to improve the detection capability. The light sources need to be collimated and the emission spectrum needs to be measured for potential qualitative and quantitative analysis. Moreover, the uniformity of illumination should also be considered to avoid the degradation of image quality due to changes in light and shade [44].

### 2.3. Sensors

Reliable sensing systems play an important role in underwater investigations of UHI. In underwater scenes, the real color of underwater images can be ‘filtered’ by the water column, and complex conditions may cause image distortion. The role of sensors is to acquire data for calibration.

Sensors equipped for UHI can be classified into two types. One is used to measure the optical properties of water, typically focusing on the light attenuation caused by absorption and scattering. In addition to the absorption of water itself, the absorption and fluorescence emission of the composition in water should not be ignored. For deep-sea or clear water areas, there is usually little or no chlorophyll in the water column. However, for near-shore areas, enrichment of chlorophyll and colored dissolved organic matter (CDOM) will change the spectrum reflected from underwater objects. An assessment of the impact of the dissolved organic matter can be performed by a fluorescence sensor or other related sensors [45]. Debris, suspended matter, and impurities also make a valuable contribution to optical scattering. Therefore, the scattering coefficient can be calibrated by a measurement of the concentration of suspended particles [46].

Another type of sensor is mainly used for monitoring the behavior of underwater vehicles. UHI for large-scale mapping is usually designed as a scanning system, allowing it to move back and forth at a height of several meters from the seafloor. During the acquisition, the parameters of height, attitude, and speed measured by an altimeter and attitude sensor should be monitored and recorded. Navigation and positing data can be obtained through dead reckoning, long or short baseline systems. By gathering data with these parameters, a navigation dataset is created for geometric correction and geocorrection in the hyperspectral image cube.

## 3. Consideration of the Water Column’s Influence in Image Processing

Eliminating the influences of absorption and scattering is a special consideration for UHI applications. The absorption coefficient of water is quite different for optical wavelengths, which is low in the blue-green wave band and much higher in near-infrared and ultraviolet wave bands. Except for water, most CDOM and suspended particles in water exhibit absorption and fluorescence emission [47], also affecting the measurement accuracy of the reflectance spectrum. Moreover, forward scattering and backward scattering caused by water molecules, CDOM, and particles lead to a large number of stray lights and decrease the contrast and clarity of hyperspectral images. In an underwater environment, therefore, it is a challenge for hyperspectral imaging to obtain the correct reflectance spectrum of objects due to absorption and scattering of the water column.

In general, several methods can be applied for correction. For underwater areas without prior knowledge, there are two solutions: one is to try to place the hyperspectral imager at a very close range from the underwater objects, so that the water influence can be ignored approximately [48]. This is not recommended in practical applications, because of the low accuracy and the sacrifice of the observation range. The other solution is to use a diffusing plate with a known reflectance. By placing the diffusing plate close to underwater objects to be observed, the reflectance characteristics of objects can be measured through comparing the normalization of the gray-scale value in each pixel of hyperspectral images [49]. This method is pretty convenient for laboratory research, although it ignores the distribution of the radial underwater light field. For practical seafloor mapping, it is unrealistic to place the diffusing plate on the seabed in real time; thus, another approach has been proposed to fix the diffusing plate on underwater vehicles [24]. Therefore, the reflectance characteristics can be estimated by measuring the distance between the underwater vehicles, diffusing plate, and seabed, respectively.

**Figure 2:**
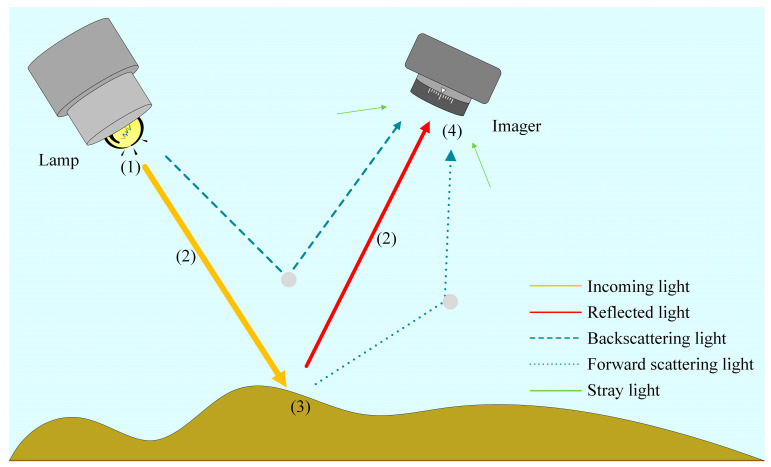
Underwater imaging model: The main processes are (**1**) radiation of the light source, (**2**) light propagation in the water column, (**3**) reflected after incident on the target surface, and (**4**) receiving and imaging by the imager.

The methods for being close to the objects or placing the standards are only suitable for the correction of underwater stationary objects. Hence, modeling methods have been proposed to meet the needs of UHI for large-scale scanning. This kind of model mainly includes the simulation of the properties of the light source; the light propagation in water; and the reflection of objects based on geometrical optics, radiative transfer theory, and the Lambert-Beer’s law. A concise underwater imaging model is shown in Figure 2.

An underwater imaging model usually includes a consideration of the radiation properties of the light source, light propagation, reflection process, and imaging process. The real color of underwater targets, that is, the reflectance of objects in hyperspectral images, can be obtained through inverse solutions from the collected images combined with the properties of the water column and light source. For the radiation of the light source, Schubert et al. [50] studied the spectral irradiance composition and short-term irradiance fluctuations caused by vertical mixing and wave focusing in shallow water under solar illumination. In the case of an artificial light source, Kolagani et al. [51] modeled the light source as a point source and studied the distribution of the underwater light field at different distances. Guo et al. [52] analyzed the axial underwater light field of LED in air and water based on a point source model, and the difference between the fitting results and the actual light source was only 1.15%.

Absorption and scattering always exist in the propagation of light, which can be estimated by the radiative transfer theory or Lambert-Beer’s law. In addition, Mortazavi et al. [53] proposed a filter based on the Oakley-Bu Cost Function to detect the optical backscattering in every spectral band from a multispectral image cube. This method could compensate for the influence of optical backscattering in water with a different turbidity without water parameter information.

When analyzing the reflection of light, the object surface is commonly assumed to be a Lambertian surface, on which light reflection occurs in all directions equally. The intensity of the reflected light depends on the diffuse reflectance of the objects, which means that the collected images can be correlated with the reflectance of the objects. Moreover, the effect of vignetting and the influence of the optical glass window between the water and imager also need to be considered. Bryson et al. [54] introduced the three-dimensional topography of the seafloor into the underwater imaging model to reconstruct the real color of underwater objects from the collected data. Sørensen [55] simulated the reflectance from underwater objects by using the Oran-Nayar reflectance model, and simultaneously studied the influence of uneven illumination on the image classification accuracy through normalization and estimating reflectance approaches. Guo et al. [56] presented a model for accurately restoring the brightness of underwater spectral images based on the underwater multispectral imager (UMI) with a filter wheel, and calibrated the model coefficients with spectral images captured underwater and in air. The refraction influence caused by the optical glass window in front of the camera was also considered in this model.

A simulation based on a modeling method needs the support of the optical properties of the water column, such as the attenuation coefficient, scattering influence, and upward and downward irradiance. In some studies [57,58], computer models were used to simulate the formation of underwater images. By inputting the configuration of the camera, light source, and water parameters, the final image output could be generated through computing. Bongiorno et al. [59,60] also presented a different model for color correction, based on the measurement of upward and downward irradiance. Combined with the stereo Red-Green-Blue (RGB, color mode) camera fixed on the autonomous underwater vehicle (AUV), in-situ hyperspectral mapping and reconstruction of the reflectance spectrum can be realized. In addition to these correction methods, some approaches based on machine learning and image-based modeling can also be used for underwater hyperspectral image processing [61,62]. The modeling methods are usually based on several assumptions and the information of the water column. To some extent, more introduced parameters of the water column may improve the accuracy of correction, but also significantly raise the complexity of calculation.

## 4. Applications of Underwater Hyperspectral Imaging Technology

### 4.1. Marine Mineral Exploration

There are abundant unexploited mineral resources in deep-sea areas, including manganese nodules, cobalt-rich crusts, polymetallic sulfides, and so on. A manganese nodule is a collection of iron oxides and manganese oxides, typically exposed or half-buried in the seafloor sediment at a depth of 4000–6000 m [63]. Cobalt-rich crusts contain abundant strategic cobalt which is lacking on the land and they are found on seamounts, ridges, or plateaus at a depth of 800–2500 m [64]. Polymetallic sulfide contains cuprum, lead, aurum, argentum, and other metallic resources. It is mainly found on mid-ocean ridges, rift valleys, and hydrothermal vents, with a depth of around 1500–3700 m [65]. The reserves of all these minerals are astonishing. However, the exploration of minerals is extremely limited in deep-sea environments. The methods employed for mineral exploration commonly include in-situ sampling, seismic, transient electromagnetics, sonar surveying, underwater photography, and spectroscopy [66,67,68,69,70].

The use of a hyperspectral imager for mineral exploration and mapping through airborne or ground platforms is well-documented [71], but it is still a challenge to conduct underwater mineral exploration by using UHI. The classification of onshore minerals mostly depends on the spectral features in short- and long-wavelength infrared bands; however, the transmission of infrared radiation is markedly inhibited in the water column. Therefore, the identification of minerals only relies on features in visible bands. Some surveys have been carried out by using the UHI deployed on underwater vehicles for exploring the minerals on the seabed. Current studies on marine mineral exploration by UHI are shown in Table 2.

Johnson et al. [24] used UHI to obtain optical fingerprints of manganese nodules, kelp, corals, and other artificial samples, and found that manganese nodules have recognizable features in the normalized reflectance spectrum. Then, according to different classification methods, various objects of interest (such as manganese nodules, rusted pieces of iron, and multicomponent substrates) were identified using UHI.

After that, Sture et al. [72] surveyed sulfide mineral deposits at the Atlantic Mid-Ocean ridge by using an underwater hyperspectral imager deployed on an AUV. The investigation area was located in Loki’s Castle at a depth of 2350 m. An underwater hyperspectral imager with a wider spectral range and four 130W LEDs were used to obtain more spectral features in near-infrared bands. The hyperspectral images processed by pseudo RGB reconstruction and principal component analysis showed the difference in sulfide deposits. Moreover, this work demonstrated that it is possible to deploy a hyperspectral imager on an AUV for seafloor minerals mapping.

**Figure 3:**
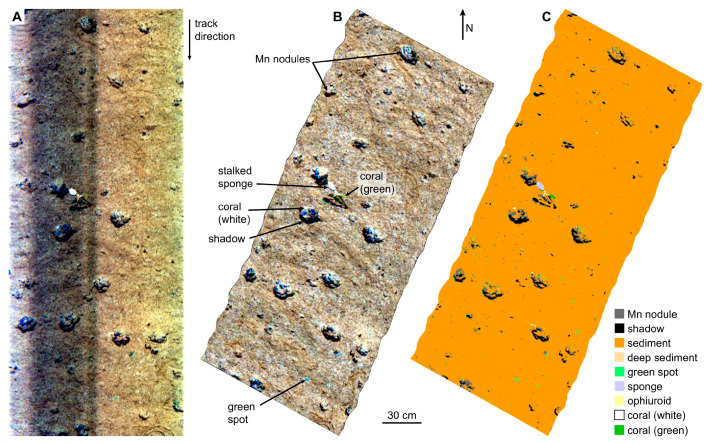
Left to right: (**A**) UHI radiance data in Red-Green-Blue (RGB) (R: 645 nm, G: 571 nm, B: 473 nm); (**B**) geocorrected pseudo-reflectance data in pseudo-RGB, and (**C**) support vector machine (SVM) classification image based on the data in (**B**). Dumke et al. [25]. Copyright (2020), with permission from Elsevier.

Dumke et al. [25] reported a hyperspectral imaging survey of deep-sea manganese nodules in 2018. The hyperspectral data was obtained by underwater hyperspectral imager deployed on a remotely operated vehicle (ROV) through the Peru basin nodules mining area at a depth of 4195 m in the southeast Pacific. Due to the inevitable changes in pitch, roll, and yaw angles of the ROV and the limitation of the accuracy of underwater positioning and navigation, there is a certain impact on underwater hyperspectral images. However, the negative effects are well corrected by using the median-spectra approach and fine georeferencing to create the reference spectra. On account of a lack of understanding of lighting conditions and the inherent optical properties of the water column, the actual result is pseudo-reflectance data. Two supervised classification methods were used and the results showed that the support vector machine (SVM) method was superior to the spectral angle mapper (SAM) method in manganese nodules. The RGB image, the pseudo-RGB image based on geometric correction, and the SVM classification image can be clearly seen in Figure 3. According to the results of SVM classification images, the coverage rates of manganese nodules in the investigated areas were calculated, varying between 1% and 9% due to many manganese nodules being buried under the sediments in some areas.

Later, in order to avoid the negative impact caused by underwater vehicles during hyperspectral imager acquisition, Dumke et al. [73] proposed a new configuration for UHI. The hyperspectral imager was deployed on a fixed platform (lander) and placed on the seafloor while the hyperspectral data could be acquired by swinging the UHI device. Compared with previous systems, this one sacrificed the imaging range in exchange for stable and high-quality images. Similar to the previous study on manganese nodules, the median spectra approach and SVM classification method were used in this study. Multiple hydrothermal and non-hydrothermal substances and benthos were found based on the modified systems in the Trans-Atlantic Geotraverse hydrothermal field at the Mid-Atlantic Ridge at water depths of 3530–3660 m. Because the pseudo-reflectance spectra cannot give the true for some underwater objects, the material composition of these objects cannot be identified. However, different hydrothermal and non-hydrothermal materials were still distinguished by SVM classification. These findings demonstrated the possibility of employing UHI for detecting different types of seafloor materials in hydrothermal areas.

In previous studies on manganese nodules and hydrothermal materials, the hyperspectral data were nearly pseudo-reflectance spectra processed by the median-spectra approach, making it difficult to reflect the real components of minerals and perform subsequent quantitative analysis. Sture et al. [74] proposed an underwater light propagation model to calculate the absolute reflectance spectra of the underwater objects. The model ignored the effects such as diffraction, wave interference and the secondary emission of photons from the target material and aimed to remove the interference irrelevant to the reflective properties of the target in a final step. Then, the model was used to correct the reflectance spectra of the underwater hyperspectral images of four kinds of substrates taken from hydrothermal vents in a laboratory tank, including low-grade seafloor massive sulfide (SMS), basalt, high-grade SMS, and mudstone. A de-noising method was also introduced to effectively control the noise of the spectral edge. Therefore, combined with the actual reflectance spectra, four substrates were identified with unsupervised dimensionality reduction. In addition, some suggestions for the UHI configuration and operation were given in this article, which may be used as a reference for field applications of mineral exploration.

Thus far, there have been few reports on research of deep-sea mineral exploration by using UHI, which is still in the exploratory stage and needs more in-depth studies. However, judging from the current achievements, this technology is still encouraging. For actual deep-sea mining, cost is a factor to be considered, thus it is necessary to find out the grade and approximate reserves of the deposits. Some challenges regarding UHI in mineral exploration need to be solved. For example, ultraviolet and infrared light are almost unavailable, caused by attenuation of the water column; therefore, many minerals lack recognizable and qualitative features in the visible band. Sometimes, sediments with a complex composition and nonuniform distribution may confuse the identification of targets. More sensitive sensors and deeper-lever spectral analysis methods such as spectral unmixing may help in solving these problems. Moreover, optical fingerprint databases of mineral deposits with different types and contents should be established for accuracy classification and identification.

### 4.2. Benthic Habitat Mapping

As one of the most important ecological types on the earth, marine ecosystems are being threatened. Overfishing, deep-sea mining, sea reclamation, and oil spilling are exacerbating the degradation of benthic habitats and accelerating the extinction of species. The potential risks need to be managed and the continuous monitoring of benthic habitats and biota is required. In shallow water areas, hyperspectral remote sensing has been widely used to identify and monitor the health status of shallow water organisms, such as corals, seagrass, and algae, as well as to send early warnings of harmful algae bloom [75,76,77]. Nevertheless, for the deep-sea, passive hyperspectral remote sensing is no longer applicable. Current methods at disposal for obtaining descriptions of seafloor benthic habitats and organisms are sonar surveying, underwater photography, and grab or trawl sampling [78,79,80]. Sonar surveying is more concerned with the topographical features of benthic habitats. Underwater sampling may not always be a good choice because of the difficulty of sampling, and it often results in the injury and death of biological samples (even high-fidelity sampling). However, UHI as a classification tool offers the possibility of the in-situ observation of organisms and benthic habitats. UHI for applications of marine organisms’ research and benthic habitat mapping has been demonstrated in several examples, which are shown in Table 3.

#### 4.2.1. Laboratory Study of Marine Organisms

Early studies of marine organisms conducted by using UHI were based on the analysis of biological samples obtained by underwater sampling. In some studies, the reflectance spectrum of marine organisms was considered to be related to the pigment composition and absorption properties in their bodies [88]. Therefore, in order to verify whether UHI can be used as a taxonomic tool for marine species according to spectral signatures, Pettersen et al. [81] extracted pigments from four benthos (spoonworm *Bonellia viridis*, sponges *Isodictya palmata*, *Hymedesmia paupertas*, and *Hymedesima* sp.) for analysis by using liquid chromatography, liquid chromatography–mass spectroscopy, and nuclear magnetic resonance. The analysis results showed that the absorbance spectrum of extracted pigment is negatively correlated with the hyperspectral reflectance spectrum for the studied benthos. Hence, a spectral database can be built for the automatic classification of pigmented organisms in marine based on the specific optical fingerprints.

Mogstad et al. [82] used a similar method to analyze the spectral characteristics of different species of coralline algae. Coralline algae pigments such as R-phycoerythrin and chlorophyll were identified by using spectrophotometry, high-performance liquid chromatography, and UHI. The results showed that different species of coralline algae were difficult to distinguish due to the similarity of the reflectance spectrum. However, in the investigation of shallow water areas, the supervised classification of coralline algae illustrated that coralline algae as a group can still be distinguished from other objects.

Some attempts of using UHI for health monitoring and ecological assessment in marine environments have also made some progress. Letnes et al. [84] exposed cold-water corals samples to the toxic compound 2-methylnaphthalene solution in concentrations of 0 mg∙L^−1^ to 3.5 mg∙L^−1^, and then classified the samples with different health statuses by machine learning based on their hyperspectral reflectance spectrum and lethal concentration levels. All samples were classified correctly and the results indicated that UHI has the potential to be used in the health monitoring of marine organisms and the assessment of environmental deterioration.

#### 4.2.2. Shallow Water Benthic Habitat Mapping

**Figure 4:**
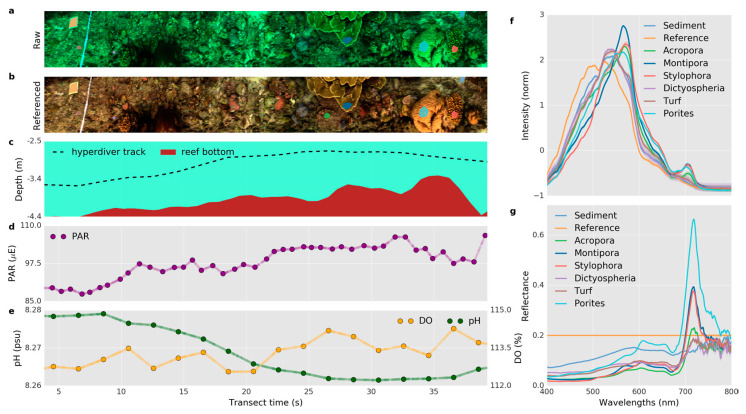
A multi-faced dataset from HyperDiver. (**a**) Three-channel color image; (**b**) a true-color image after correction; (**c**) depth and altitude information; (**d**) the photosynthetically active radiation (PAR) intensity; (**e**) dissolved oxygen and pH over the transect; (**f**) the average spectral intensity; and (**g**) the spectral reflectance of targets. Reprinted from Chennu et al. [36].

Seagrass and coral reefs are typical ecosystems in shallow water areas. Seagrass systems are important in providing habitats and food for other marine life and keeping the water clean. Similarly, coral reefs are of extreme economic value, while protecting the coast and maintaining biodiversity. Chennu et al. [36] designed HyperDiver for benthic habitat mapping. HyperDiver can collect quite comprehensive information, including hyperspectral images, RGB images, and several parameters of the water column. Figure 4 shows the high-level information on the underwater environment provided by HyperDiver. HyperDiver can cover 15–30 m^2^ of scanning areas per minute with the pushing of scuba divers. Machine learning was introduced to map the seagrass, coral reefs, and benthic species in a shallow water habitat at a centimeter spatial resolution, and the accuracy reached up to 93–97%. In the following study, Rashid and Chennu [89] annotated 47 class labels up to genus- and species-levels for massive amounts of data collected by HyperDiver, which benefited the automatic classification of objects in interest in underwater hyperspectral images.

Mogstad et al. [85] attempted to deploy the underwater hyperspectral imager on an unmanned surface vehicle (USV) for shallow water habitat mapping. USV was easy to be deployed and handled, and it could be operated in shallower areas, which are usually inaccessible to AUV and ROV. The hyperspectral data of survey areas covering 176 square meters were obtained at a depth of 1.5 m in a sheltered bay. Six objects of interest, such as coralline algae, green algae films, and invertebrates, were identified, and the SVM classification accuracy was about 90% based on confusion matrix analysis. Such a deployment of an underwater hyperspectral imager may be more suitable for investigation of the underwater environments in near-shore or calm waters.

Cimoli et al. [87] creatively developed an under-ice hyperspectral imager to observe the sea-ice at Cape Evans, Antarctica, from the particular “inverted” under-ice prospective. The hyperspectral imager was deployed in an under-ice sled system for scanning the bottom of sea-ice in push-broom mode. Different from the coarse point sampling of ice, the hyperspectral imager could provide a high spatial resolution, which reached up to a sub-millimeter level. As a non-invasive method, it provided a new way to observe biological features and quantify the chlorophyll concentration of sea-ice.

#### 4.2.3. Deep-Sea Survey

**Figure 5:**
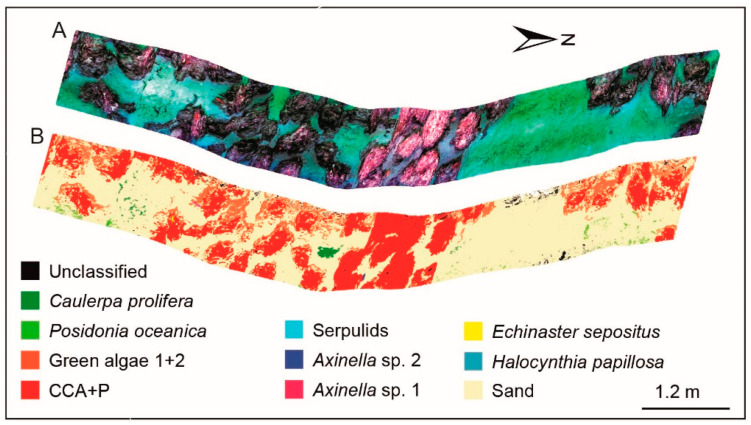
Cold-water coral habitat mapping: (**A**) RGB image of the coralligenous site, and (**B**) spectral angle mapper (SAM) classification image based on data in (**A**). Reprinted from Foglini et al. [86].

Deep-sea ecosystems are of great significance in the study of species’ origin and evolution and geochemistry. Recently, Foglini et al. [86] presented a survey on cold-water corals in the Southern Adriatic Sea at a depth of 200–700 m. By using UHI, several biological groups (such as *Madrepora oculata*, *Desmophyllum pertusum*, *Desmophyllum dianthus,* and large fan-shaped sponges) in this area were identified and classified. One of the classification images is shown in Figure 5. The optical fingerprints of species were introduced to classify different species of cold-water coral habitats based on SAM classification. This study showed that UHI can realize the quantifiable and repeatable mapping of habitats according to comprehensive spectral libraries.

Large organisms in the deep-sea have always attracted the attention of biologists and oceanographers. A survey indicated that deep-sea experts generally believed large organisms (that is, macro- and megafauna) should be given priority in the monitoring of deep-sea benthic habitats due to their important role [90]. Due to the complexity of deep-sea sampling, Dumke et al. [83] discussed the possibility of employing UHI directly as an in situ taxonomic tool for deep-sea megafauna. With the reference spectrum and supervised classification, 30 types of different targets (various megafauna, including bony fish, sponges, ophiuroids, and so on) were classified in manganese nodules field in the Peru basin at a depth of 4200 m based on the difference of the reflectance spectrum. These discoveries proved that many organisms that were difficult to be distinguished in RGB images were certain in hyperspectral images, and the complex operation of the physical collection of samples in deep-sea areas could perhaps be replaced by UHI.

Marine ecosystems are very fragile. Once damaged, it often takes several years or even longer for them to recover. Habitat degradation and recovery rates have been identified as crucial features for monitoring deep-sea ecosystems, and they are also applied to entire marine ecosystems. Marine organisms may have more recognizable spectral characteristics than minerals in visible bands, which may make UHI more flexible in benthic habitat mapping. Up to now, UHI has demonstrated its potential as a taxonomic tool for in situ marine investigation, and is expected to be used in ecosystem health assessment. Further trends may promote the application of UHI in the study of marine primary productivity and marine biodiversity, and provide strategies for marine management under artificial measures.

### 4.3. Underwater Archaeology and Pipeline Inspection

**Figure 6:**
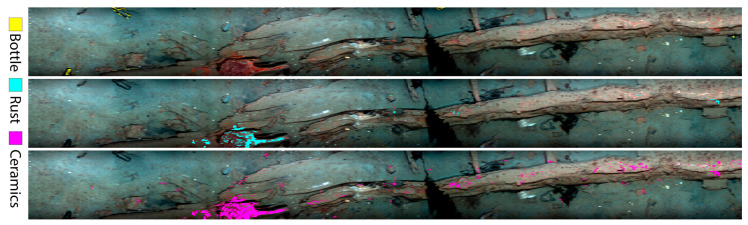
Supervised SAM classification images of a wreck transect. From top to bottom: Glass bottle, rust, and ceramics. Picture is rotated 90° clockwise. Reprinted with permission from Ødegård et al. [27]. © The Optical Society.

Underwater archaeology is one of the potential applications of UHI. Underwater archaeology is the interdiscipline of archaeology and marine science, which is of great significance to the study of ancient history and overseas communications history. Underwater archaeology is mainly employed to investigate and excavate underwater cultural heritages such as sunken ships and ancient sites. Almost all underwater cultural heritages can be found near the main channel of human shipping activities or coastal waters. In 2016, UHI was used in a survey of shipwreck sites by Ødegård et al. [91]. The underwater hyperspectral imager was deployed on ROV, and the scanning lines of ROV and the investigation plan were determined with the support of a sonar and underwater high-definition video camera. Additionally, the information of shipwreck sites, including underwater hyperspectral images, was obtained for underwater archaeological research. After that, Ødegård et al. [27] selected ceramics, metals, glass bottles, and wood, which often appeared in underwater cultural heritage sites, and measured their optical fingerprints by using UHI in a laboratory. Then, these optical fingerprints were used to classify the objects in situ at the shipwreck sites. The supervised SAM classification images of wreck transect are shown in Figure 6. Although there were some errors in the classification of objects, it was still a meaningful research that explored the feasibility of UHI in underwater archaeology.

**Figure 7:**
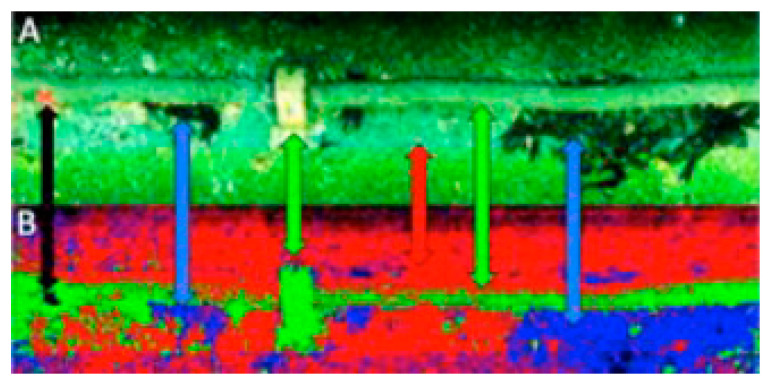
Underwater pipeline inspection: (**A**) False RGB image of a pipeline and surrounding objects, and (**B**) unsupervised classification image based on the data in (**A**). Reprinted from Johnsen et al. [92]. Acknowledgement of © 2016 International Federation of Automatic Control.

Advances in marine mineral exploration and benthic habitat mapping have demonstrated the potential of UHI in underwater target detection, but as a novel taxonomic tool, ambitious researchers are still not satisfied with the capabilities of UHI and are trying to broaden the application scope of UHI technologies. Johnsen et al. [92] introduced the potential application of UHI in pipeline inspecting. The underwater false RGB image (A) and the unsupervised classification image (B) of the pipeline are shown in Figure 7. Red, green, blue, and black in the underwater hyperspectral image respectively represent the sand/soft bottom, concrete pipeline, brown algae dominated by kelp, and starfish. It can be seen that the underwater hyperspectral imager provided more intuitive information compared to underwater cameras. It has been reported that Ecotone is developing a UHI instrument for underwater pipeline monitoring, and this can be used to detect corrosion or leakage which is invisible to conventional methods.

## 5. Discussion and Conclusions

**Figure 8:**
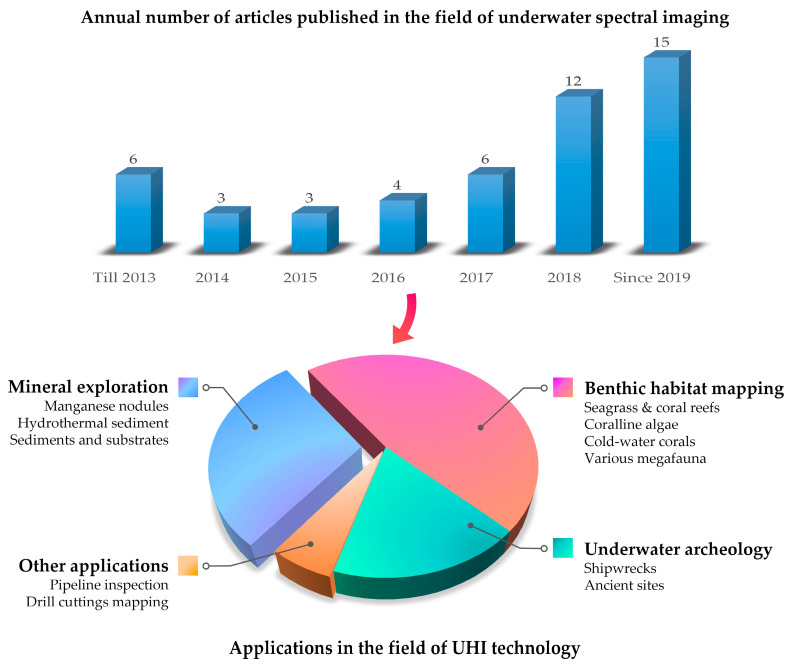
The development trend of underwater spectral imaging technology. Top picture: Statistics on the number of articles in the field of underwater spectral imaging technology in recent years. Bottom picture: Current applications in the field of UHI technology.

The seafloor is rich in information, thus, what we need to do is get as much data from it as possible. UHI offers a new perspective for seabed observation. In this way, organisms, species, objects, and sediments can be identified and mapped in detail. In recent years, underwater spectral imaging technology has gradually attracted attention. Figure 8 presents the development trend of underwater spectral imaging technology and the applications in the field of UHI technology in recent years. Most UHI applications have focused on habitat and sediment mapping in shallow and deep-sea areas; however, there are a few other applications that have explored the potential of UHI in the field of underwater archaeology, pipeline inspection, and seafloor drill cuttings survey [93]. Generally speaking, UHI has more advantages in some specific scenes. UHI is more flexible than spaceborne and airborne hyperspectral imaging, thus, it can reach some difficult-to-access areas and capture images with a fairly high resolution. Therefore, for some shallow and coastal areas, UHI is expected to be used in marine fisheries and aquaculture to monitor the changes in food, waste, and seabed composition. It also may be used for the investigation of changes and occurrences in coastal ecological environments. Due to the complex environment of deep-sea areas, the further usage of UHI needs to be explored. However, UHI can still be used as an automated classification tool to provide high-quality data for potential applications.

In practical underwater applications, the complexity of the underwater environment and the stability of underwater vehicles limit the development of UHI technologies, resulting in a large gap between the hyperspectral imaging technologies that are applied underwater or in air. In addition, the strategic value in military and economy fields means that the sharing of UHI technologies is greatly limited. There are multiple architectures of the underwater spectral imaging system, which exhibit great differences in underwater applications. Therefore, it is extremely important to select the appropriate spectral imaging system for different scenes. The most basic aspects to be considered are: (1) active or passive imaging, depending on whether it is used in shallow water or in deep-sea; (2) the expected spectral and spatial resolution; (3) the imaging speed and size of the investigation area; and (4) the cost and budget. Table 4 presents a comparison of different architectures of the underwater spectral imaging system according to its typical spectral splitter, spectral resolution, imaging speed, cost, and difficulty of geometric correction.

The push-broom underwater spectral imaging system can achieve a high spectral resolution, but geometric correction is still a problem, which needs the support of inertial navigation and other auxiliary systems. In order to ensure the quality of hyperspectral images and reduce the difficulty of image mosaics, a higher accuracy in attitude control and better stability of underwater vehicles are needed. A push-broom system is able to achieve a wide range of coverage in investigation area at a slow cruising speed. This makes the push-broom architecture very suitable for seabed mapping in relatively flat areas and large-scale habitat investigations, such as those on coral reefs and seagrass beds.

Due to the spectral-splitting modes, most of the staring underwater spectral imaging systems are multispectral systems, which have a low spectral resolution and slow imaging speed. However, the staring system is more suitable for the observation of stationary objects by placing the hyperspectral camera on the seabed or fixing it on the hovering underwater vehicles. Using the filter wheel as the spectral splitter may be the preferable choice of most staring systems at present because of the simple design and optional number of imaging bands, but systems with moving parts may not be the best solution for underwater applications. With the help of underwater cameras, many staring systems can easily realize geometric correction for underwater hyperspectral images. Moreover, the staring system can set the exposure time of each band separately so that the entire hyperspectral image cube has a higher dynamic range.

The snapshot underwater spectral imaging system has a high resistance to external interference in underwater environments and high-throughput. Additionally, the snapshot system, by way of its data acquisition, which can obtain images from the whole spectral bands by a single exposure [94], may become a new trend in future underwater spectral imaging technologies. The complex system assembly and data post-processing may need to be solved with the continuous development of hyperspectral imaging technologies. More advanced and miniaturized UHI sensors will be a constant pursuit for a period of time. Future UHI sensors may be designed for unmanned underwater platforms, so as to reduce the complexity of deployment during operation and expand the scope of scanning. Certainly, the selection of underwater spectral systems should also be considered according to the needs of practical applications, and the consideration of cost is also a factor that cannot be ignored in underwater operations.

At the moment, the investigation technologies of underwater targets are also devoted to diversification development. Emerging sensors and solutions have enriched the selection of underwater operations. Table 5 presents a comparison of the difference of various underwater survey technologies including conventional acoustic imaging and optical methods.

Overall, it is quite clear that no single acoustic or optical method fits all of the underwater observation and detection needs, and none of them can cover very different ranges and resolutions. However, what is interesting is that there are a few examples that have begun to explore underwater applications through the combination of sonar detecting, three-dimensional (3D) imaging, UHI, and other technologies [95,96,97]. The combination of multiple technologies may be a trend of underwater target detection in the future, in which UHI technology could provide a possibility for a secondary inspection of the interested targets or areas observed by acoustic methods. On the other hand, the limitations of each technology will be overcome through the use of UHI, in combination with other technologies. Further analysis of UHI data and the fusion of data from multiple sensors will become a new research direction of UHI. Although this is beyond the scope of this paper, it will help to promote UHI technology’s use in a wider range of applications.

Finally, as previously mentioned, UHI technologies provide a possibility to observe and map the underwater world. There are not many reports on UHI technologies, but a few commercial systems are currently available. According to the specific optical fingerprints of the objects, UHI has been able to identify and classify substrates such as minerals, sediments, benthos, and underwater remains. Naturally, challenges exist, but the influence of the water column and others can be corrected in some methods. Further work can be expected to establish a closer relationship between the underwater hyperspectral data and the characteristics of the targets, and to combine the UHI with other underwater sensors, such as light detection and ranging (LiDAR), 3D imaging sensors, synthetic aperture sonar sensors, and polarizing cameras to create more informative underwater descriptions. In addition, UHI technologies have the potentially to be used in the military, so as to realize the inspection of underwater channels and identification of underwater camouflage targets such as mines.

## Figures and Tables

**Table 1:** Some of the underwater spectral imaging systems. LUMIS: low-light-level underwater multispectral imaging system; UMSI: underwater spectral imaging system.

Model	Developer	Spectral Range/Bands	Resolution	Spatial Imaging	Depth Rating
LUMIS	Zawada [29]	460,522,582,678 nm/4	12.0–42.1 nm		20 m
UMSI	Wu et al. [31]	400–700 nm/31	10 nm	Staring ^2^	50 m
TuLUMIS	Liu et al. [32]	400–700 nm/8	>10 nm	Staring	2000 m
UHI OV ^1^	Ecotone	380–750 nm/150–200	2.2–5.5 nm	Push-broom ^3^	6000 m
U185	Cubert Gmbh	450–950 nm/125	8 nm@532 nm	Snapshot ^4^	5 m
WaterCam	Sphere Optics	450–950 nm/138	8 nm@532 nm	Snapshot	

^1^ UHI Ocean Vision; ^2^ Staring: wavelength scan; ^3^ Push-broom: line scan; ^4^ Snapshot: single shot.

**Table 2:** Examples of marine mineral exploration conducted by using UHI.

Summary	Platforms	Materials	Methods	Reference
A comprehensive introduction to UHI, and observation and classification of objects placed manually on the seafloor by a UHI-carrying cart.	Underwater cart	Substrates, minerals, animals	Field	Johnsen et al. [24] (2013)
The first time a full-scale hyperspectral imager has been mounted on an autonomous underwater vehicle (AUV) for massive sulfide deposit mapping.	AUV	Sulfide deposits	Field	Sture et al. [72] (2017)
First underwater hyperspectral imaging in-situ survey of manganese nodules in the Peru Basin (SE Pacific Ocean) at about a 4195 m water depth.	ROV ^1^	Manganese nodules	Field	Dumke et al. [25] (2018)
Mapping of the Trans-Atlantic Geotraverse (TAG) hydrothermal field by using UHI deployed on a stationary platform that classified several hydrothermal materials.	Lander	Hydrothermal materials	Field	Dumke et al. [73] (2019)
A methodology for calculating the reflectance and strategies for noise mitigation were proposed to recover spectral signatures for the classification of materials.	/	Massive sulfide deposits	Lab	Sture et al. [74] (2019)

^1^ ROV: Remotely operated vehicle.

**Table 3:** Examples for applications of UHI in organism and benthic habitat surveys.

Summary	Platforms	Areas	Objects	Methods	Reference
Study on the species-specific absorption and hyperspectral reflection signatures of marine organisms by pigment extraction.	/	/	Spoonworms, sponges	Lab	Petterson et al. [81] (2014)
The application of HyperDiver operated by a diver in the investigation of tropical coral reefs.	Diver	Shallow water	Benthic habitat	Field	Chennu et al. [36] (2017)
Spectral characteristics of coralline algae obtained by pigment extraction for habitat investigation.	ROV	Shallow water	Coralline algae	Lab, Field	Mogstad et al. [82] (2017)
UHI as a taxonomic tool for the in-situ observation of deep-sea megafauna.	ROV	Deep-sea	Deep-sea megafauna	Field	Dumke et al. [83] (2018)
Observation and classification of samples exposed to 2-methylnaphthalene to evaluate the ability of UHI in coral health monitoring.	/	/	Cold-water corals	Lab	Letnes et al. [84] (2019)
Feasibility study on an attempt of benthic habitat mapping by using UHI deployed on USV in shallow areas.	USV ^1^	Shallow water	Benthic habitat	Field	Mogstad et al. [85] (2019)
Underwater habitat mapping of cold-water in the Mediterranean Sea based on spectral libraries of several objects.	ROV	Deep-sea	Cold-water coral habitat	Field	Foglini et al. [86] (2019)
Non-invasive inverted system with a particular perspective to observe ice algae and calculate the chlorophyll concentration.	Under-ice sled	Under-ice	Sea ice	Field	Cimoli et al. [87] (2019)

^1^ USV: Unmanned surface vehicle.

**Table 4:** Comparison of different architectures of the underwater spectral imaging system. LCTF: liquid crystal tunable filter.

Type	Spectral Splitter	Spectral Resolution	Imaging Speed	Geometric Correction	Price
Push-broom	Prisms, gratings	High	High	Difficult	High
Staring	Filter wheel	Intermediate	Low	Easy	Low
	LCTF	Intermediate	Low	Easy	Intermediate
	Tunable LED	Low	Intermediate	Easy	Low
Snapshot	Prisms, gratings, filters	Low	High	Easy	High

**Table 5:** Comparison of different underwater survey technologies.

Sensors	Technology	Range	Bathymetry	Imagery	Spatial Resolution	Price
MBS ^1^	Acoustic	<11,000 m	√	√	Low	High
SSS ^2^	Acoustic	<150 m	√	√	Intermediate	High
SAS ^3^	Acoustic	<260 m	√	√	Intermediate	High
LiDAR	Optical	~tens of meters	√	×	/	High
Video	Optical	<10 m	×	√	High	Low
Raman or LIBS	Optical	Very close	×	×	/	High
UHI	Optical	<10 m	×	√	High	High

^1^ MBS: Multibeam bathymetry; ^2^ SSS: Side scan sonar; ^3^ SAS: Synthetic aperture sonar.
